# How, when, and who should ask about pregnancy intentions in primary care? A qualitative study of women’s preferences

**DOI:** 10.1093/fampra/cmad114

**Published:** 2023-12-20

**Authors:** Jennifer A Hall, Kira Wilkinson, Claire Haddon, Geraldine Barrett

**Affiliations:** UCL EGA Institute for Women’s Health, Reproductive Health Research Department, Sexual and Reproductive Health Research Team, London, United Kingdom; Public Participant, United Kingdom; Public Participant, United Kingdom; UCL EGA Institute for Women’s Health, Reproductive Health Research Department, Sexual and Reproductive Health Research Team, London, United Kingdom

**Keywords:** contraception, preconception care, pregnancy intention screening, primary care, reproductive intentions, reproductive health

## Abstract

**Background:**

For health services to help people plan for or prevent pregnancy, health professionals need an acceptable way to identify individuals’ preferences.

**Objective:**

To assess women’s views on the acceptability of specific questions about pregnancy preferences when asked by health professionals in a variety of primary care contexts.

**Methods:**

One-to-one in-depth interviews with 13 women aged 18–48 from across the UK, involving role-play scenarios and ranking exercises. Interviews covered a range of settings and health professionals, different question wording, and ways of asking (in person or digitally). We conducted a thematic Framework Analysis, focussing on themes relating to feelings and preferences.

**Results:**

Women were generally open to being asked about pregnancy preferences if they understood the rationale, it was asked in a relevant context, such as in women’s health-related consultations, and there was follow-up. After signposting, an open question, such as ‘How would you feel about having a baby in the next year?’ was preferred in a face-to-face context as it enabled discussion. While some women valued a face-to-face discussion with a health professional, for others the privacy and convenience of a digital option was preferred; methods should be tailored to the target population.

**Conclusion:**

Discussion of pregnancy preferences via a range of formats is acceptable to, and valued by, women in the UK across a range of primary care settings. Acceptability to health professionals and feasibility of implementation needs further exploration and would benefit from greater public awareness of the benefits of pregnancy planning.

**Key messages**

- Decisions about pregnancy planning and prevention are complex and sensitive.
- Health professionals often report concerns about how best to approach this topic.
- Discussion of pregnancy preferences in primary care is acceptable to women.
- Face-to-face and digital formats are required to meet different groups’ needs.
- Greater public awareness of the benefits of pregnancy planning would enable this.

## Introduction

‘When are you going to have children?’ There is a general sense that it is sensitive, personal, and possibly prohibited to ask people if they are thinking about or are trying for a pregnancy.^1^ However, for services to be able to meet people’s needs, whether to avoid unplanned pregnancies through appropriate contraception or to prepare for pregnancy by improving general health, healthcare professionals need to find an acceptable way of asking people what their plans are.^2^ Following the 2018 Lancet Preconception Health Series,^3^ a 2019 strategy called for a dual approach to improving preconception health by focussing on both individual and the population level.^4^ Key aims within the individual strategy were to ‘normalise conversations about planning for pregnancy’ and ‘to improve identification of people who are planning a pregnancy’.^4^ The latter of these is a particular barrier to providing preconception health advice^5,6^ but could be achieved through pregnancy intention screening in primary care.^7,8^ Building on previous work,^2^ a new model of integrated contraception and preconception care highlighted the need to consider reproductive needs across the lifecourse and that the entry point of pregnancy intention screening is key to that.^9^

A 2019 review showed pregnancy intention screening to be generally acceptable.^10^ Specifically, patients in the USA found the topic highly acceptable, with only 1.8% of women, 4.3% of men, and 6.8% of transgender/other patients not wanting to be asked about their reproductive intentions.^11^ Several different ways of raising the topic of pregnancy have been explored, including a reproductive life plan,^12^ the One Key Question (OKQ) approach,^13^ the psychometrically validated Desire to Avoid Pregnancy (DAP) Scale,^14^ the Pregnancy Attitudes, Timing, and How important is pregnancy prevention (PATH) questions,^15^ or screening for family planning service needs.^7,16^

There have been few direct comparisons of these approaches, and often no clear preference among patients, making it challenging to know what to implement. One survey asked over 1,000 patients in federally funded primary care in New York State about four questions, one of which was the OKQ.^11^ Around one third of patients had no preference but among patients expressing a preference, a question in a clinical encounter asking about reproductive service needs was preferred across men, women, and transgender patients (26.6%, 33.1%, and 36.4%, respectively).^11^ Another comparison involving the OKQ found no preference between the options presented though it was a small study (*n* = 84).^17^ However, current research has been conducted in the USA, where populations and service structures are very different, limiting generalisability of the findings to the UK.

In 2018 the Pregnancy Planning, Prevention, and Preparation (P3 Study) recruited 1000 people who were female sex at birth and of reproductive age from across the UK.^18,19^ At baseline, participants were asked several questions about pregnancy preferences, including one similar to the OKQ, and the DAP scale.^14^ The DAP is a 14-item scale, designed for self-completion in a written format, with response options on a five-point Likert scale from Strongly Agree to Strongly Disagree.^14^ It was developed to prospectively measure women’s preferences and feelings about a potential pregnancy, and how strongly they wish to avoid pregnancy. The DAP was evaluated for use in the UK,^18^ found to be predictive of pregnancy,^19^ and compared with other ways of asking about pregnancy preferences.^20^ A shortlist of questions for potential clinical use was developed based on their confirmed ability to identify whether someone is likely to become pregnant, to give the clinician the confidence to tailor their clinical advice accordingly. The aim of this study is to assess women’s (participants self-reported as female; here we refer to ‘women’ and this should be taken to include people who do not identify as women but who have the capability to become pregnant) views on the acceptability of the shortlisted DAP questions when asked by health professionals in clinical contexts, and to explore the option of a digital tool containing these questions.

## Methods

To explore women’s views, we conducted one-to-one qualitative in-depth interviews, our methodological approach closely aligned to a qualitative description.^21^ We emailed 225 women from the P3 Study who had agreed to be contacted about further research and who, at the time of enrolment in 2018, did not have an under- or postgraduate degree. This was to ensure that our sample would not over-represent highly educated women. Forty-two responded, 18 were invited to interview, purposively selected to ensure a range of ages, ethnicities, and pregnancy histories. Inclusion criteria were those implemented in the P3 Study; females aged 15 or over who were pre-menopausal; not sterilized; and living in the UK. This strategy was employed to ensure a maximum-variation sample and increase the information power of each interview.^22^

### Data collection

The interview topic guide was informed by the two online discussion groups, where 12 women discussed a range of scenarios where they might be asked about pregnancy preferences. Women reported that they were willing to talk about pregnancy preferences in a variety of settings, but expressed strong reservations about being asked in pharmacies,^9^ mainly due to privacy concerns, so we excluded pharmacies.

As we were asking women to consider how they might feel about being asked about pregnancy preferences, essentially hypothetical situations rather than actual experiences, we were conscious of including in the topic guide a range of ways for interviewees to engage with the subject. Role play, with the interviewer playing the part of a healthcare professional (HCP) asking the pregnancy preference questions, was used to explore women’s reactions and opinions. Cognitive interviewing techniques were employed to allow a detailed consideration of the questions and their understandability. Ranking exercises were incorporated to facilitate discussion about preferred HCPs and different ways in which questions about pregnancy preferences could be asked, including a digital format. The topic guide was revised iteratively during the first few interviews to improve the flow and to present suggestions made by earlier participants to later participants (see Supplementary material S1.) The specific wording of the questions that were taken from the DAP and put to women is shown in Box 1. No attempt was made before the interviews to adapt the questions to being asked verbally.

Box 1 **Wording of questions discussed in the interviews (questions are taken from the DAP scale).**

How much would you agree or disagree that:

It would be a good thing for you if you became pregnant in the next 3 months.

It would be the end of the world for you to have a baby in the next year.

You want to have a baby within the next year.

Thinking about becoming pregnant in the next 3 months makes you feel excited.

You would worry that having a baby in the next year would make it harder for you to achieve other things in your life.

*Five-point Likert scale ranging from Strongly agree to Strongly Disagree*

### Research team and reflexivity

The interviews were conducted via Zoom, transcribed, and anonymised by JH, a female medical doctor and Public Health researcher with training and experience in conducting interviews. She is a white, married, mother of three children with a long-standing research interest in helping people achieve their reproductive goals. Field notes were made, and transcripts were sent to participants for review at their request though none returned any comments. JH did not know the participants prior to the study; participants knew the purpose of the research before consenting.

### Data analysis

We conducted a Framework Analysis, as appropriate for applied clinical research,^23^ using Nvivo. JH developed the coding frame, creating four initial descriptive high-level codes based on the topic guide (background about the participant, thoughts or feelings about being asked, context and person, what happens next), with subcodes based on women’s responses in the interview. The coding frame was tested, discussed with GB (who had access to the original interview transcripts and provided an independent, second perspective), and then refined and applied it to all interviews in the indexing stage by JH. Charting was undertaken, using framework matrices to compare and contrast the preferences of women with different characteristics, and to consider the findings of the ranking activities. The initial findings were discussed among the research team and patient and public involvement (PPI) group, with iterative discussions throughout the analysis. We viewed the data through a realist lens, focussing on experiential themes relating to the participants’ feelings and preferences.^24^

### Patient and Public Involvement

The P3 Study PPI group reviewed and revised the information sheet, consent form, and topic guide, discussed shortlisted questions and scenarios, and gave feedback on the coding frame and findings during the analysis and on the write up.

## Results

### Sample

Of the 18 women invited to interview, 13 consented and 5 did not respond; the socio-demographics are shown in Table 1. Women fell into two groups: ‘younger women’ who were under 30, unmarried with no children (*n* = 7); and ‘older women’, who were over 30, more likely to be married and to either have been pregnant or have children (*n* = 6). Interviews lasted 46 minutes on average.

**Table 1.** Socio-demographics of the 13 participants asked about their opinions of being asked about pregnancy preferences in a range of primary care settings in the UK in 2022.

Person	Age group	Ethnicity	Relationship	Children	Location
NW001	40+	White British	Married	Yes	London
NW002	20–24	Mixed	Other relationship	No	London
NW003	20–24	Asian	Other relationship	No	Midlands
NW004	40+	Other	No relationship	Yes	London
NW005	35–39	White British	Married	Yes	South east
NW006	25–29	White British	No relationship	No	North east
NW007	35–39	White British	Married	Yes	Midlands
NW008	40+	White British	Married	Yes	Scotland
NW009	40+	White British	Married	Yes	Midlands
NW010	15–19	White British	Other relationship	No	London
NW011	20–24	White British	Other relationship	No	South west
NW012	15–19	Black British	No relationship	No	Midlands
NW013	20–24	White British	Other relationship	No	London

### Findings

**Fig. 1.**
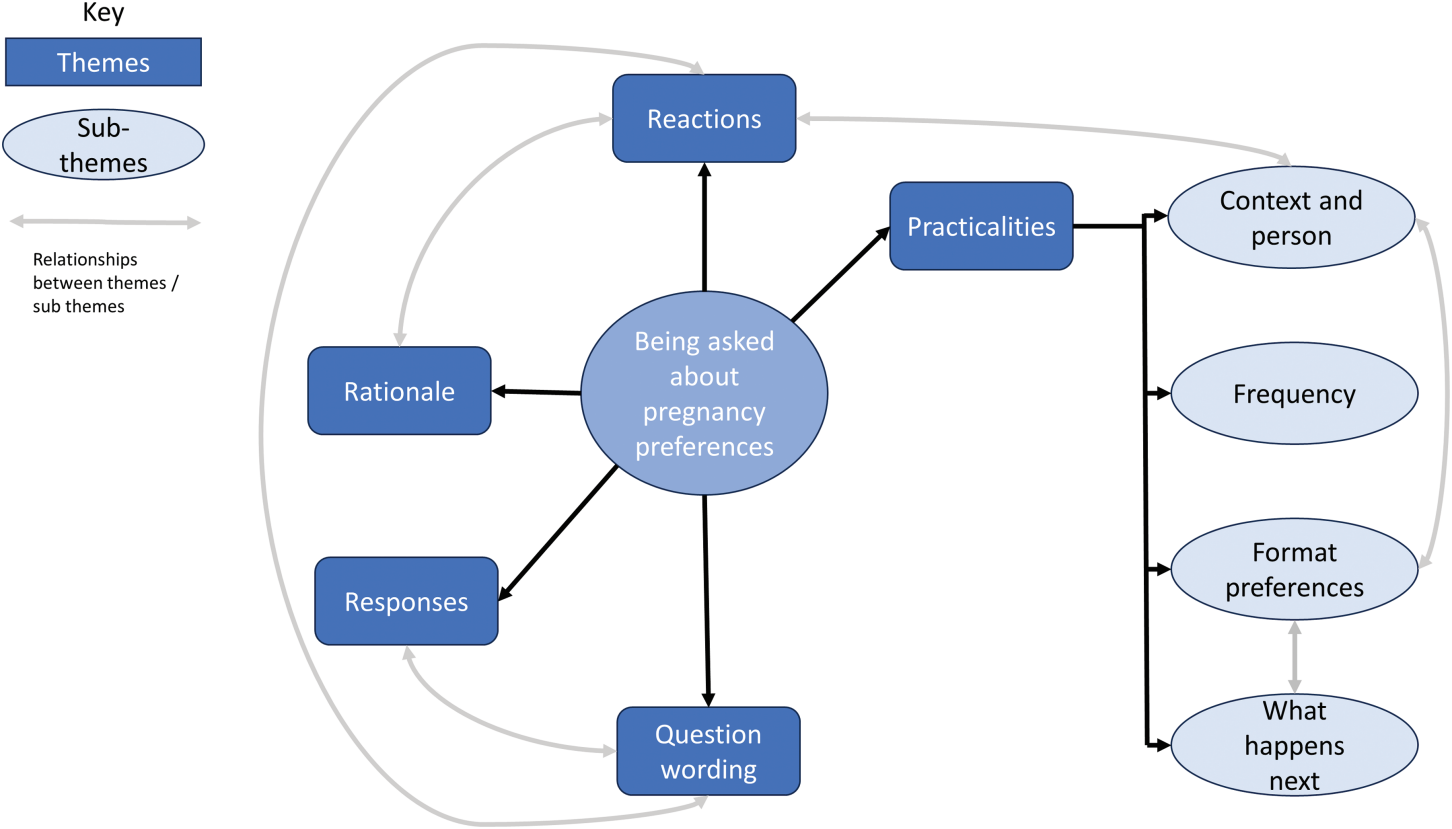
Representation of the themes and sub-themes, with the relationships between them, from women interviewed on their opinions of being asked about pregnancy preferences in the UK in 2022.

The five main themes (Rationale, Reaction, Responses, Question wording, and Practicalities) and four sub-themes of the Practicalities theme (Context and Person, Frequency, Format Preferences, and What happens next) are shown in Fig. 1. There were links between some themes; for example who and where the question was asked (context and person) influenced women’s reactions, hence some findings are presented across themes/sub-themes. Except where specific questions were asked about, findings are related to being asked about pregnancy preferences generally.

#### Rationale, reaction, and frequency of asking about pregnancy preferences

When asked why they thought an HCP might ask about pregnancy preferences, women gave a range of reasons, mostly related to the offer of support and advice, in particular to help choose the right contraceptive or bringing it up in case people are not comfortable raising the topic themselves. For example, NW009 said ‘People might not feel happy about opening up that discussion themselves, but it gives them the opportunity then to ask … you know they’ve been asked the question they feel they can answer it as opposed to feeling uncomfortable about bringing it up themselves’. Asking about pregnancy preferences was mostly seen as acceptable as part of routine lifestyle checks, which would help to normalize it. Women felt this would be a positive thing in terms of including sexual and reproductive health (SRH) within a holistic approach to health and normalizing the topic.

Outside of a woman’s health consultation, women stated that there needed to be a clear rationale for asking about pregnancy preferences, particularly while it is not common practice. Prescribing medication or the management of existing conditions were considered suitable reasons. If a rationale was not given women might try to second guess why they were being asked which could cause worry or risked irritating women ‘because … it just feels like alright, okay well that’s just how I am viewed—as a pregnancy machine’ (NW013).

It was acceptable to ask annually (or at each contact if less frequent than a year) from around the age of 15 until women were postmenopausal. Some women suggested an opt out should be available, others agreed when this was put to them. In all cases the opt out was for other groups, not the interviewee herself, such as people with fertility problems or those who knew that they did not want children. This was linked to women’s reaction; no one found being asked about pregnancy preferences upsetting themselves, but some noted it could be personal, sensitive, or even intrusive, depending on the individual and context, hence the potential need for an opt-out. Younger women talked about fear of judgement by the clinician, with regard to expectations that they should be on contraception, whereas older women generally felt more comfortable discussing pregnancy preferences. There was some discomfort about only women being asked this and the societal expectation that all women should want babies, as noted by NW013 above, which questions like this could, however unintentionally, reinforce.

### Context and person

Most women thought ‘women’s health’ and particularly contraception appointments were a reasonable opportunity to ask as ‘it feels like pregnancy is directly related to contraception’ (NW007) but NW010 wondered why she should be asked if they had gone for contraception as, clearly, she did not want to get pregnant. Others, and indeed this woman later in the interview, saw how these questions could help the HCP ‘work out what contraception you need’ (NW011) based on if/when/how far away she might want a pregnancy. For one ‘I wouldn’t bat an eyelid if they also have questions after my smear test, it’s very relevant, we’re already discussing down there’ (NW005) but for another ‘it would feel a bit personal if it was a, like a smear appointment’ (NW007).

Given that there was a strong preference for questions about pregnancy preferences to be asked at appointments that were already related to women’s health as ‘it’s a very logical tie in’ (NW005) there was a preference for professionals for whom this was their specialization. Across the professions considered, many women did not distinguish between doctors and nurses. Where they did it was frequently due to their experiences or relationships with particular people.

Overall SRH doctors or nurses were the preference: ‘I would almost expect them to ask that’ (NW007). Older women’s preference was to be asked by a practice nurse, but this was because they were less likely to access community SRH services. This was followed by practice nurses and GPs when women were seeing them for a ‘women’s health issue’ and then health visitors (a specialist community nurse who works with families with a child under 5 years old). There was least agreement about health visitors. Women who were younger or had no children were less likely to know what a health visitor was and though they thought it might be an appropriate time they also thought that women might not be receptive to this conversation when they had just had a baby. This was reinforced by the older women, several of whom said that while they would generally be happy to speak to their health visitor about this and consider interconception health, this conversation should not happen in the early postpartum period.

Some of the younger women suggested staff at educational settings such as the teacher in charge of wellbeing or school nurse, and one woman suggested that if you had had a difficult pregnancy then the obstetrician could talk to you about getting pregnant again; a midwife could also potentially do this.

### Question wording and responses

We tested an introductory sentence of ‘I ask all my patients of reproductive age about pregnancy, in case I can offer advice about contraception or preparation for pregnancy [is that ok?]’. This was widely liked as it was inclusive, minimized the feeling of targeting, signalled that the topic was coming enabling people to opt-out, and gave some explanation as to why they were being asked.

The DAP questions about whether a pregnancy would be a ‘good thing’ for them and whether they ‘wanted a baby’ in the next year were the most widely acceptable questions. They were considered inoffensive, non-judgemental, and easy to understand. Other DAP questions asking whether thinking about a pregnancy made them excited, or whether a baby would make it harder to manage other things in their lives, were more divisive. Younger women were more likely to like both, seeing the question about excitement as upbeat and about how they would manage as pertinent and woman-centred, but some found the latter question too negative. Whether a baby would be the end of the world was widely described as too emotive and negative, and not the sort of question that they would expect from a HCP. However, it was also felt to be clear and some could see how this question could be useful with regard to the choice of contraception, possibly as a follow-up if women were unsure. In this context asking more than one question was reasonable.

The DAP questions asked about pregnancy in the next 3 months or a baby in the next year. While technically the same thing, they were experienced differently by the women. The next 3 months felt much more immediate and pressured; women preferred to consider a longer time frame.

The format of asking someone verbally how much they agreed or disagreed with a statement was not felt to work as well as it does within a written survey. All women answered the questions in a way that was clear that they understood, but only four used the words ‘agree’ or ‘disagree’ anywhere in their response. Instead, women gave more detailed explanations of their current thoughts and circumstances. From women’s answers to these questions, there was a strong sense that this is a ‘complex question’ (NW011) that should be open to ‘more than just a yes or no’ (NW005). This was particularly relevant to women whose feelings were less clear cut or if they were neutral or ambivalent. Four women independently suggested variations of: How would you feel about a pregnancy/baby in the next three months/year? This was put to two women in their interviews, and they liked it.

### What happens next

Most women, when asked what should happen after these questions had been asked, expected some sort of action; discussion, advice, or signposting, and/or an opportunity to come back for further discussion. Whether women wanted a discussion there and then depended on what they already knew, what kind of appointment they were in, what the relationship with the HCP was like, and how they preferred to receive information. Recognizing this, eight women suggested that women should be offered a choice in how to proceed, for example, ‘do you want to come back and talk to me about this, or I can sign post you to these websites or this information leaflet?’ (NW009). Particularly with regard to contraception options, women thought an initial discussion followed by a chance to go away and think about it, with signposting to sources of further information, and a further appointment was valuable. Offering a choice ‘feels more empowering, more open and less judgemental’ (NW012). Women recognized that they had a need for this information, particularly younger women or those or who had not had a pregnancy before.

### Format preferences

The preference of women across all age groups was to complete the questions themselves digitally (e.g. online or in an app), complemented by having a subsequent discussion with an HCP, if needed. Digital completion was liked because it was felt to provide benefits such as autonomy, convenience, privacy, time to think, and partner inclusion. Women said that digital completion felt less awkward and that they would not feel judged, particularly if they thought their answers did not appear to make sense or were contradictory. It was acceptable for women to be directed to a website/app by an HCP, the NHS (e.g. when booking an appointment, posters) or by school/higher education.

Some women were keen for everything to be digital: ‘I think …, providing, enabling people to have the information they need takes pressure off the NHS and people can very effectively look at that’ (NW007) but for another ‘I feel like you’re not getting the right support, you’re kind of just neglected and said to just do everything online’ (NW003). The main drawbacks with digital completion and advice were that you would not have the chance for discussion, which some women valued, you cannot get personalized advice, and that there is an overwhelming amount of information online, not all of which is trustworthy. ‘I kind of find myself, just like researching the next question, the next question and all the information is coming from different websites and different sources, and then by the end of it, you know something, Google’s told you you’ve got cancer’ (NW003).

The questions being asked in person tended to be less favoured, mainly due to concerns about capacity or because women were not in contact with health services frequently. There is clearly not a one-size-fits all; as one woman summarized: ‘not getting pregnant, and monitoring my sexual health and that sort of thing feels really important and something I want to get right and something that I want to discuss and I want to discuss options … and I feel like in person it’s the best way to do it. But, I also feel like … I’m thinking of lots of my, my friends, you know, having it in that digital format makes it so much more approachable and doable for so many more women, so I do feel like that’s a really good option as well’ (NW011).

## Discussion

Overall, women in this study found it acceptable to be asked about their pregnancy preferences in primary care consultation or to complete these questions digitally, in line with other studies.^11,17,25–28^ They were happy to be asked by doctors, nurses, and health visitors in a range of primary care settings. As others have found, there was a preference for consultations that were related to women’s health,^29^ fitting with a Making Every Contact Count approach,^30^ or where there was a clear rationale, such as informing management of an existing condition or when prescribing. This aligns with evidence from women with a wide range of chronic conditions on the complexity of their childbearing decisions^31^ and desire for opportunities to discuss contraception and pregnancy options, making this discussion key to providing patient-centred care.^32–34^ In keeping with other studies,^11^ they were happy to be asked regularly, with annually acceptable.

While women in online discussions were not keen on being asked in pharmacies,^9^ as in some other studies,^35^ this does not preclude any role for pharmacists. However, consideration needs to be given to ensuring privacy, as women do not want to be ‘outed’ by accessing services.^1^ Whatever the setting, creating a safe space, where there is confidence and credibility in the questioner such that the person feels able to speak honestly and will receive meaningful advice and support, is vital. To do this it is crucial that taboos around admitting you are trying for a baby or are struggling with fertility are broken down.

The screening questions we considered, taken from the DAP scale, have Likert responses for self-completion.^14^ It is on this basis that their ability to predict pregnancy was assessed and this was used as a criterion for shortlisting them. However, most women in this study gave full, descriptive explanations when asked these questions in the role play, rather than simply how much they agreed/disagreed, suggesting more open questions are preferred. In a face-to-face clinical encounter, the value of the questions is twofold. Firstly, as a prompt to raise an important health issue that is often overlooked^17^ or de-prioritized by both patient and clinician.^1,31^ Secondly, for the HCP to be able to gauge the woman’s current orientation toward pregnancy so that they may provide the most appropriate service. The proven predictive ability of these questions,^19,20^ gives clinicians confidence that they will more accurately identify who is and who is not likely to become pregnant than questions like ‘are you currently trying to get pregnant?’ where most pregnancies would be missed^20^ or the OKQ, which has not been widely favoured.^11,36^

The tension between what women prefer (a format that allows discussion and ambiguity) and what providers need (brevity and information on which to act)^17^ could perhaps best be navigated with a ‘blended’ approach. This could combine digital and in-person modalities, relieving some of the burden from services while also supporting women. This could include a combination of: (1) routinely directing women to complete questions digitally with links to validated digital advice and information on how to arrange a follow-up discussion with an HCP if required; and (2) HCPs opportunistically asking about pregnancy preferences and signposting to digital advice, with the potential for the woman to return to ask questions, discuss further and receive contraception or preconception advice, if needed. Regardless of approach used, any discussion should be client-centred, empowering and support individuals to develop, articulate and act on their preferences in a non-judgemental, non-coercive way, while recognizing that women may not have clearly defined intentions.^15,37,38^

## Strengths and limitations

This study included women who were diverse in many ways, covering a wide age range, geographical locations, relationship status, sexuality, and a variety of fertility histories. However, they were all English-speaking women, so language or other cultural issues could not be explored, nor could particular experiences such as domestic violence. They were also all cis-gender women and none raised the issue of asking trans-men their pregnancy preferences. Since transgender and non-binary people are more likely to prefer not to be asked about pregnancy^11^ further work must explore their preferences. This was one of the groups, along with others such as those who have experienced early menopause, that the PPI group thought would benefit from an opt-out. Other studies have suggested that asking about SRH service needs may be more appropriate for this group than the narrower focus on pregnancy intention screening.^11^ Another limitation is that we asked about women’s preferences in hypothetical scenarios. Whilst we incorporated role play and other tools the preferences expressed in this study may not translate into the healthcare setting. Finally, we studied women as the questions under consideration were developed and tested only in women. To ensure that pregnancy prevention and preparation are not seen solely as ‘women’s issues’ it is vital that similar work is done with men.

## Conclusion

Our study shows that raising a discussion of pregnancy preferences within a clinical encounter is acceptable to, and even valued by, women in the UK, where they understand the rationale. There is no single correct setting, format or person, suggesting implementation across a range of modalities, in line with a recently published model of care and Making Every Contact Count approach, would be suitable. Further work should explore the acceptability to clinicians in the UK and the feasibility of implementation screening for pregnancy preferences in primary care, both digitally and in person. Prior to any such implementation, it would be important to raise awareness within the general population of why they may be asked such questions by their health professionals, as well as making digital tools available for those who wish to self-manage these aspects of their reproductive health. This approach was strongly supported by the PPI group who felt there was a lack of openness and awareness around fertility and reproductive health and that this could help de-mystify it and make it more socially acceptable to discuss. Further work should be done to explore the acceptability of asking everybody of reproductive age about pregnancy preferences, regardless of sex, gender identity, or sexuality, and supporting choice, whether that is to avoid pregnancy or not, as this would support a population level shift in attitudes towards the management of SRH.

## Supplementary data

Supplementary data is available at *Family Practice Journal* online.

cmad114_suppl_Supplementary_Material

## Data Availability

The data underlying this article cannot be shared publicly as we do not have consent to place transcripts in a public repository. The anonymised transcripts will be shared on reasonable request with the corresponding author.
