# Supplementary material for: How, when, and who should ask about pregnancy intentions in primary care? A qualitative study of women’s preferences
Source: Fam Pract. 2023 Dec 20;41(2):131–8. doi: 10.1093/fampra/cmad114 (PMC11017777; doi:10.1093/fampra/cmad114)
Supplement: cmad114_suppl_Supplementary_Material [file cmad114_suppl_supplementary_material.pdf]

Supplementary materials

S1 – Topic guide

S2 – COREQ checklist

Topic guide For Interviews with Women

UCL Research Ethics Committee Approval ID Number: 3974/003

Thank you again for agreeing to take part in this research. I am Dr Jennifer Hall and I am leading this research.

As explained in the information sheet, we are interested in **testing different ways of exploring people's feelings and preferences regarding a future pregnancy**. We have previously tested a range of questions in an online survey and we have narrowed the questions down to a few which could potentially be used to identify who would like to become pregnant in the next year and who would not. We also explored people's preferences for how they would like to be asked and by whom, if anyone.

I'd like to take you through a series of activities to explore your preferences about some of the questions and ways in which they could be asked that we have put together based on the research so far. Please be as critical as you want; the purpose of these interviews is to develop plans to suit people like you. There are three main sections to the interview: first we will focus on the specific questions; second on the context and person asking the questions; and finally on what you would like to happen next.

Ultimately, the hope is that these questions will help people clarify what they want in relation to pregnancy. This will also help health professionals to identify who needs advice about contraception, and who needs advice about preparing for pregnancy.

This interview is confidential and once it has been transcribed and any identifying details have been taken out, the (video) recording will be deleted. I may still make some notes while you are talking but I am still listening to you.

Do you have any questions before we start?

Confirm happy to continue.

BEGIN RECORDING

Before we start to discuss the questions, please could you tell me a little bit about yourself? It would be useful to hear a bit about you, your background and whether you have ever been pregnant or have any children.

Background

- Check we know a bit about her, and prompt if needed, in terms of

- Age
- Relationship status
- Obstetric history
- location
- Employment (primarily medical or not)

That's great, thanks.

QUESTIONS

As I was saying, first I would like to discuss the specific questions that you could be asked. We tested a number of different questions and have narrowed it down to a few different options, and it is this that we would like your opinion on.

For now, I would like you to imagine that you are seeing your practice nurse for a women's health issue – something like contraception or your cervical smear for example. I'm going to ask a question and I would like you to think about how you would answer that question, and how you would feel about being asked it.

During the consultation the nurse says;

I ask all my patients of reproductive age about pregnancy, in case I can offer advice about contraception or preparation for pregnancy is that ok? How much do you agree or disagree that: It would be a good thing for you if you became pregnant in the next 3 months.

How would you feel about being asked that question? Is there anything you particularly liked or disliked? Why?

What did you think the reasons were that the nurse was asking this question?

I am going to share my screen with you and on it you will see three different questions. We are considering using one, two or all three of these questions. Take your time, read through them, feel free to answer the questions, either out loud or in your head, but you don't need to tell me your answers if you don't want to.

The questions under consideration are:

It would be a good thing for me if I became pregnant in the next 3 months.

Strongly Agree	*Agree*	*Neither agree nor disagree*	*Disagree*	*Strongly Disagree*
□	□	□	□	□

It would be the end of the world for me to have a baby in the next year.

Strongly Agree	*Agree*	*Neither agree nor disagree*	*Disagree*	*Strongly Disagree*
□	□	□	□	□

I want to have a baby within the next year.

Strongly Agree	*Agree*	*Neither agree nor disagree*	*Disagree*	*Strongly Disagree*
□	□	□	□	□

- Is there anything about any of the questions that you particularly liked or disliked? Why?
- Do you have any other thoughts on these questions?
- How would you feel about answering one, two or all three of these questions?
- How many feels acceptable?

Now I am going to show you some other possible questions – the first one is the same and the second two are different. Once you have read them, please talk me through your thoughts.

Which question, or combination, do you think would result in the most useful conversation from your point of view as a patient?

In this scenario, what did you think about starting the question with something like 'I ask all my patients of reproductive age…' does it make it better / worse / no difference?

Any other comments or suggestions on the questions?

How could you see these questions being asked in a health care setting, if at all?

SETTING
Now I'd like you to look at this set of options and think about being asked your preferred question(s) in these scenarios. Take your time to read them all and talk me through your preference, as in where you could be most comfortable being asked about your thoughts about a future pregnancy. By 'women's health issue' we mean something like contraception, cervical screening, consultation for menstrual issues etc.

Scenarios:

- Seeing/ speaking to your General Practitioner (GP) for a women's health issue
- Seeing/ speaking to your General Practitioner (GP) for another health issue
- Seeing/ speaking to your Practice nurse for a women's health issue
- Seeing/ speaking to your Practice nurse for another health issue
- Seeing/ speaking to a Sexual and Reproductive Health doctor (eg at the family planning or 'GUM' clinic)
- Seeing/ speaking to a Sexual and Reproductive Health nurse (eg at the family planning or 'GUM' clinic)
- Seeing/ speaking to a health visitor after you have had a baby
- Seeing/ speaking to a someone else
- I would not like to be asked by anyone, I'd rather complete them myself

Could you tell me a bit about how you came to your order of preference?

Discuss how they ranked them, why, any strong preferences.

Does your preference change if the healthcare professional was male?

Has your preference changed across age?

FOLLOW UP

Now I would like to talk about what you would like to happen after you have been asked the question(s). What would you expect to happen next?

How would you feel if the health care professional used your answer to direct you to an online source of information, such as a website to help you choose a method of contraception / plan a pregnancy?

Instead of being asked by a health care professional (or if they say they do not want to be asked), how would you feel about being directed to an 'app' or website that would take you through these sorts of questions?

Would you like the same app/ website to give you advice based on your answers?

How likely would you be to use this sort of app or website without a health care professional suggesting it, for example if a friend recommended it or you saw an advert for it?

I am going to share my screen again, with four different options for you to consider. Which of these would you prefer and why?

In terms of format, how would you rank these four options:

1. Completely digital format (that is you answer questions and receive advice in an app / on a website)
2. To be asked your preferred question(s) by a health care professional and be directed to digital advice
3. To complete your preferred question(s) yourself in a digital format and then discuss your answers with a health care professional (in person or remotely)
4. For all of it to be done in person

RLP 'app'

We are considering an app or website that would support people to achieve their reproductive goals throughout their life, whether avoiding or preparing for pregnancy. The idea is that you would be asked your preferred question(s) as an entry point to the app and depending on your answers you would then proceed towards either contraception advice and links to services or to an assessment of your preconception health, again with links to advice and services.

How does this sound to you? What would you want from an app like this?

Do you have any other comments on when, who or how you would find it acceptable to address these issues?

End of interview

Thanks so for much for your time. That’s all my questions and I really appreciate your input. Do you have any questions?

OK, so just a bit of admin to wrap up.

- Ask if they would like to receive a copy of the transcript
- **Stop recording**.
- Ask if there is anything that they would like to discuss privately
- Tell participant how they will receive their voucher.
- Reflexivity: Write up relevant observational notes from the interview.

Notes

COREQ (COnsolidated criteria for REporting Qualitative research) Checklist

A checklist of items that should be included in reports of qualitative research. You must report the page number in your manuscript where you consider each of the items listed in this checklist. If you have not included this information, either revise your manuscript accordingly before submitting or note N/A.

Topic	Item No.	Guide Questions/Description	Reported on Page No.
**Domain 1: Research team and reflexivity**			
Personal characteristics			
Interviewer/facilitator	1	Which author/s conducted the interview or focus group?	6
Credentials	2	What were the researcher's credentials? E.g. PhD, MD	6
Occupation	3	What was their occupation at the time of the study?	6
Gender	4	Was the researcher male or female?	6
Experience and training	5	What experience or training did the researcher have?	6
Relationship with participants			
Relationship established	6	Was a relationship established prior to study commencement?	6
Participant knowledge of the interviewer	7	What did the participants know about the researcher? e.g. personal goals, reasons for doing the research	6
Interviewer characteristics	8	What characteristics were reported about the inter viewer/facilitator? e.g. Bias, assumptions, reasons and interests in the research topic	6
**Domain 2: Study design**			
Theoretical framework			
Methodological orientation and Theory	9	What methodological orientation was stated to underpin the study? e.g. grounded theory, discourse analysis, ethnography, phenomenology, content analysis	6
Participant selection			
Sampling	10	How were participants selected? e.g. purposive, convenience, consecutive, snowball	4-5
Method of approach	11	How were participants approached? e.g. face-to-face, telephone, mail, email	4-5
Sample size	12	How many participants were in the study?	4,5,7
Non-participation	13	How many people refused to participate or dropped out? Reasons?	7
Setting			
Setting of data collection	14	Where was the data collected? e.g. home, clinic, workplace	6
Presence of non-participants	15	Was anyone else present besides the participants and researchers?	6
Description of sample	16	What are the important characteristics of the sample? e.g. demographic data, date	7 and table 1
Data collection			
Interview guide	17	Were questions, prompts, guides provided by the authors? Was it pilot tested?	5,7 and S1
Repeat interviews	18	Were repeat inter views carried out? If yes, how many?	no
Audio/visual recording	19	Did the research use audio or visual recording to collect the data?	6
Field notes	20	Were field notes made during and/or after the inter view or focus group?	6
Duration	21	What was the duration of the inter views or focus group?	7
Data saturation	22	Was data saturation discussed?	no
Transcripts returned	23	Were transcripts returned to participants for comment and/or	6

Topic	Item No.	Guide Questions/Description	Reported on Page No.
		correction?	
**Domain 3: analysis and findings**			
Data analysis			
Number of data coders	24	How many data coders coded the data?	6
Description of the coding tree	25	Did authors provide a description of the coding tree?	fig 1
Derivation of themes	26	Were themes identified in advance or derived from the data?	6
Software	27	What software, if applicable, was used to manage the data?	6
Participant checking	28	Did participants provide feedback on the findings?	6, 7
Reporting			
Quotations presented	29	Were participant quotations presented to illustrate the themes/findings? Was each quotation identified? e.g. participant number	8-12
Data and findings consistent	30	Was there consistency between the data presented and the findings?	8-14
Clarity of major themes	31	Were major themes clearly presented in the findings?	8-12
Clarity of minor themes	32	Is there a description of diverse cases or discussion of minor themes?	8-12

Developed from: Tong A, Sainsbury P, Craig J. Consolidated criteria for reporting qualitative research (COREQ): a 32-item checklist for interviews and focus groups. *International Journal for Quality in Health Care*. 2007. Volume 19, Number 6: pp. 349 – 357

Once you have completed this checklist, please save a copy and upload it as part of your submission. DO NOT include this checklist as part of the main manuscript document. It must be uploaded as a separate file.
